# Relation of the Older Practitioner to the Younger

**Published:** 1882-06

**Authors:** 


### RELATION OF THE OLDER PRACTITIONER TO THE YOUNGER.

[Remarks of Dr. L. W. Brophy, M.D., D.D.S., at the Banquet of the Michigan Dental Association, in Detroit, March 31, 1882.]

Mr. Chairman and Gentlemen:—From time immemorial young men of ambition, in all vocations in life, have earnestly endeavored to emulate the record of their most honorable and worthy seniors. In consequence of this zealous devotion of young men to their calling, we live in an age of progress unprecedented in history. So numerous, indeed, have been the improvements in the various departments of agriculture, commerce, manufactures, art, and science, that even the keenest observer and most assiduous student, on inspecting them, stands amazed at the marvellous achievements of man. Our own vocation, gentlemen, is second to none in this regard. The influence exerted by the older practitioners of high standing, over the younger, has been, I believe, more potent for good than most of us realize. On the other hand, the influence of incompetent, evil-minded practitioners over the young lead the latter into endless trouble, disappointment, and humiliation. How well do I remember when, twenty-two years ago, in my twelfth year, a dentist came to my father's house in the country, remained with us a week, and attended to our teeth. His beautiful pearl-handled instruments, his bright, shining gold foil, and his delicate manipulations, fascinated me so that in fancy they were ever before my eyes, and the subject of my dreams. I then resolved some day to become a dentist. Finally my father moved to the city, and through the intercession of a friend, I secured a place with a dentist who assured me that in two years he would teach all that was necessary to enable me to be an efficient practitioner. Accepting his advice, I entered upon my pupilage. After the lapse of two years I was induced to commence practice; during the three succeeding years I did my best, and was successful in the opinion of my friends. I was unable, however, to practice dentistry satisfactorily to myself. When patients came to me who had been under the care of

Corydon, Palmer, Dwinelle, Taft, Allport, and other distinguished operators, no one can realize how insignificant I felt, and how eager I was to be able to insert fillings in imitation of theirs. It was then I fully appreciated that I had been ill-advised by my preceptor, and that he should have influenced me to attend college, instead of urging me to commence practice. The character of the operations of the gentlemen whose names I have mentioned made a profound and indelible impression on my mind.

Making the acquaintance of a dental college graduate, who told me of the eminent professors and the opportunities afforded for study and clinical instruction in dental colleges, I decided to relinquish practice to go and acquire the knowledge which I so greatly needed and desired. Here I met older practitioners whose influence over young men lifted their burdens, relieved them of their difficulties and imbued their minds with the indispensable principles which underlie successful dental practice. There are, however, professional men, both young and old, I regret to say, who invariably speak derogatorily of their fellow practitioners. The young man sometimes says to his patient, on inspecting the dental operations in his mouth, "Who inserted these filling?" The patient responds, "Dr. S." "What Dr. S. That old fogy? Why, he does not use the electric plugger; he never attended dental college, in fact. he is so old he cannot see." The older practitioners' opinion is asked of the young dentist, who has recently graduated, returned from college and located on the opposite corner. In response he says, "Well, the young man has had no experience, he is filled with new fangled notions, in fact, he is all feathers." It is impossible to find language sufficiently strong to condemn such allegations. Gentlemen, if we expect to be *honored* and *respected* by the public we *must honor* and *respect* one another.

To the zealous laborers in the interest of dental advancement—the pioneers of dentistry of the West, many of whom are gathered around this festive board—whose lives have been devoted to the elevation of our calling, to the alleviation of human suffering and to the establishing of our institutions of learning, we bow in greatful acknowledgement of their eminently valuable, ennobling and honored lives. And we trust that their future pathways may be made smooth and pleasant by the willing appreciative hands of all whose good fortune it has been to know them. Who has not listened intently and profited by the wise council of the experienced, skillful and revered gray haired practitioner, who relates the cause of his early failures, his struggle to overcome difficulties and his ultimate success?

What young dentist has not inspected with feelings of unbounded enthusiasm the model dental operations, the beautiful, highly finished fillings, with well defined margins, inserted by our eminent senior brothers ?

On the other hand, the young practitioner, full of vigor and enterprise, pursuant to the wishes and aided by his elder brother, goes forward determined to merit the confidence of the public, urge and labor for a modification of the teaching of dentistry so as to surely make it a branch of the great tree of medical science, and thereby elevate it to the position which eventually it is destined to attain.

